# Making Sense of Theories, Models, and Frameworks in Digital Health Behavior Change Design: Qualitative Descriptive Study

**DOI:** 10.2196/45095

**Published:** 2023-03-15

**Authors:** Paula Voorheis, Aunima R Bhuiya, Kerry Kuluski, Quynh Pham, Jeremy Petch

**Affiliations:** 1 Institute of Health Policy, Management and Evaluation Dalla Lana School of Public Health University of Toronto Toronto, ON Canada; 2 Centre for Data Science and Digital Health Hamilton Health Sciences Hamilton, ON Canada; 3 McMaster Health Forum McMaster University Hamilton, ON Canada; 4 Institute for Better Health Trillium Health Partners Mississauga, ON Canada; 5 Centre for Digital Therapeutics Techna Institute University Health Network Toronto, ON Canada; 6 Telfer School of Management University of Ottawa Ottawa, ON Canada; 7 Division of Cardiology Faculty of Health Sciences McMaster University Hamilton, ON Canada; 8 Population Health Research Institute Hamilton Health Sciences Hamilton, ON Canada

**Keywords:** behavioral science, behavior change, health behavior, digital health, mobile health, theories, models, frameworks

## Abstract

**Background:**

Digital health interventions are increasingly being designed to support health behaviors. Although digital health interventions informed by behavioral science theories, models, and frameworks (TMFs) are more likely to be effective than those designed without them, design teams often struggle to use these evidence-informed tools. Until now, little work has been done to clarify the ways in which behavioral science TMFs can add value to digital health design.

**Objective:**

The aim of this study was to better understand how digital health design leaders select and use TMFs in design practice. The questions that were addressed included how do design leaders perceive the value of TMFs in digital health design, what considerations do design leaders make when selecting and applying TMFs, and what do design leaders think is needed in the future to advance the utility of TMFs in digital health design?

**Methods:**

This study used a qualitative description design to understand the experiences and perspectives of digital health design leaders. The participants were identified through purposive and snowball sampling. Semistructured interviews were conducted via Zoom software. Interviews were audio-recorded and transcribed using Otter.ai software. Furthermore, 3 researchers coded a sample of interview transcripts and confirmed the coding strategy. One researcher completed the qualitative analysis using a codebook thematic analysis approach.

**Results:**

Design leaders had mixed opinions on the value of behavioral science TMFs in digital health design. Leaders suggested that TMFs added the most value when viewed as a starting point rather than the final destination for evidence-informed design. Specifically, these tools added value when they acted as a *gateway drug* to behavioral science, supported health behavior conceptualization, were balanced with expert knowledge and user-centered design principles, were complementary to existing design methods, and supported both individual- and systems-level thinking. Design leaders also felt that there was a considerable nuance in selecting the most value-adding TMFs. Considerations should be made regarding their source, appropriateness, complexity, accessibility, adaptability, evidence base, purpose, influence, audience, fit with team expertise, fit with team culture, and fit with external pressures. Design leaders suggested multiple opportunities to advance the use of TMFs. These included improving TMF reporting, design, and accessibility, as well as improving design teams' capacity to use TMFs appropriately in practice.

**Conclusions:**

When designing a digital health behavior change intervention, using TMFs can help design teams to systematically integrate behavioral insights. The future of digital health behavior change design demands an easier way for designers to integrate evidence-based TMFs into practice.

## Introduction

### Background

The number of digital health interventions that aim to facilitate changes in health behaviors is rapidly increasing [1]. Digital health interventions offer the ability to deliver targeted health support to more individuals when they need it the most [2,3]. Despite the potential of digital health interventions to positively affect health, there is mixed evidence on whether these interventions are effective in changing health behaviors or improving health outcomes [3,4]. Currently, the development of digital health interventions is fragmented, with little coordination or consensus on best-practice design approaches [5,6]. A large systematic review found that digital health interventions designed with the support of behavior change theories, models, and frameworks (TMFs) are more likely to be effective than those without them [7]. Nonetheless, other studies suggest that digital health interventions are often designed without the use of these evidence-informed TMFs [1].

The delineation between TMFs has been described elsewhere; however, they overlap considerably when used in applied practice [8]. Theories typically help establish a set of relationships between variables with specific predictions [9]. For example, the Theory of Planned Behavior suggests that attitudes, subjective norms, and perceived behavioral control shape an individual’s behavioral intentions, which in turn shape behavior [10]. Models usually deliberately simplify a phenomenon or aspects of a phenomenon [8]. For instance, the Transtheoretical Model posits that individuals move through 6 general stages of behavior change [11]. Frameworks usually outline a structure of descriptive categories, concepts, or constructions [8]. For example, the Theoretical Domains Framework summarizes 14 domains that are suggested to influence behavior [12].

During the design of digital health behavior change interventions, TMFs can be advantageous in helping designers ensure that their innovations are grounded in the science of human behavior [7]. Although several popular apps such as Noom (Noom Inc) and Headspace (Headspace Health) claim to be founded on behavioral science insights, applying TMFs, such as the Capability, Opportunity, and Motivation–Behavior model, it remains unclear exactly how TMFs were used [13,14]. A recent scoping review by our authors found that digital health design teams that used behavioral science TMFs often failed to describe exactly how TMFs were integrated into their design processes [15]. Currently, no TMF or a combination of TMFs has proven to be superior for designing effective digital health interventions [15]. Design teams appear to face difficulties in selecting and using TMFs to ensure that their digital health products effectively support healthy behavior [15].

**Figure 1:**
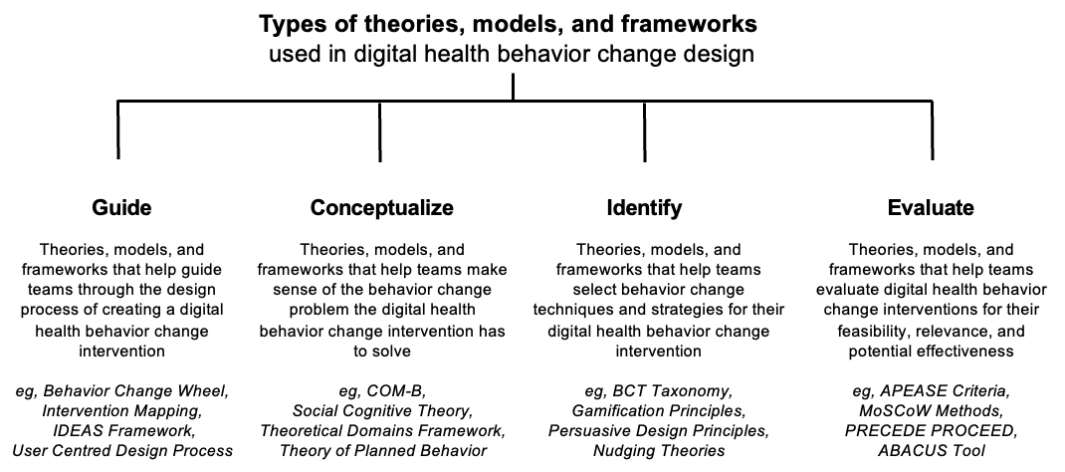
Types of theories, models, and frameworks used in digital health behavior change design (adapted from Voorheis et al [15]). ABACUS: App Behavior Change Scale; APEASE: Acceptability, Practicability, Effectiveness, Affordability, Side-effects, and Equity; BCT: behavior change technique; COM-B: Capability, Opportunity, and Motivation–Behavior; IDEAS: Integrate, Design, Assess, and Share; MoSCoW: must-have, should-have, could-have, and won't-have, or will not have right now; PRECEDE: Predisposing, Reinforcing, and Enabling Constructs in Educational Diagnosis and Evaluation; PROCEED: Policy, Regulatory, and Organizational Constructs in Educational and Environmental Development.

Clarifying the ways in which TMFs are used in digital health behavior change design may help optimize digital health design methods. In our previous scoping review, we attempted to describe the different ways TMFs are being used by design teams, which include using TMFs to (1) guide the design process itself; (2) conceptualize the behavior change problem; (3) identify relevant behavior change content and features; and (4) evaluate ideas for their applicability, feasibility, or potential effectiveness [15]. This conceptualization is illustrated in Figure 1. Although this delineation provides a helpful framework for categorizing different types of TMFs in digital health behavior change design, it does not provide any reflection or guidance on which TMFs may work best in what situations and how. To better understand the nuances of how TMFs are being used in digital health behavior change design, we need to understand how designers extract value from TMFs in practice. A lack of description on how TMFs are being used to support design makes it challenging to understand how and why a digital health intervention may have succeeded or failed, restricting our ability to identify design methods that will increase digital health intervention success.

### Aims and Objectives

The aim of this study was to explore how digital health design leaders select and use TMFs in practice. Specifically, we aimed to answer the following three questions: (1) How do design leaders perceive the value of TMFs in digital health design? (2) What considerations do design leaders consider when selecting and applying TMFs in their digital health design work? and (3) What do design leaders think is needed in the future to advance the utility of TMFs in digital health design?

## Methods

### Approach

This study uses a qualitative description approach to explore and describe the experiences and perspectives of digital health design leaders [16,17]. A qualitative description approach is relevant for gathering information about a specific phenomenon and staying close to the data to offer a summary of that phenomenon of the everyday language of participants [16]. Qualitative descriptions are drawn from the tenants of naturalistic inquiry, in which researchers commit to studying a phenomenon through the meanings that participants ascribe [17]. Our commitment to naturalistic inquiry is consistent with our philosophical and theoretical assumptions concerning the nature of reality (ontology) and knowledge (epistemology) [18]. Specifically, we embody a relativist ontological position that acknowledges that reality is created by participants’ subjective understanding of it [19,20]. The ontological position of naturalistic research such as qualitative description is fundamentally relativist and rooted in recognizing and describing that reality is subjective and varies from person to person [17]. We also embrace constructivist and pragmatist epistemological perspectives, which are aligned with their recognition that knowledge is socially constructed by participants and researchers [21,22]. Constructivist and pragmatist perspectives can be sensibly combined when researchers aim to explore and describe different participant perspectives in applied settings in relation to a practical research aim [21]. Ultimately, this research is descriptively and practically oriented, which aligns with the following two main elements of qualitative description: (1) learning from the participants and their descriptions and (2) using this knowledge to influence future practice and interventions [17]. Although we embrace social constructivism in this paper, we recognize that we use certain analytical approaches that align with a more positivist paradigm, such as multiple research coding and codebook development [23]. These deviations are justified by our pragmatic aim to describe participant perspectives in relation to our research questions and our *codebook thematic analysis* approach, which Braun and Clarke [23] suggest sits somewhere in between a positivist and subjectivist orientations [24]. Although further described in our data analysis section, *codebook thematic analysis* is a pragmatic thematic analysis approach used in applied research, which allows a more structured process to coding to meet predetermined informational needs, while also allowing researchers to respect the multiple realities of participants and use reflexive practices [24].

### Research Team

The research team included 5 researchers affiliated with the University of Toronto. The lead researcher is a PhD candidate (PV; she/her) whose research aims to explore digital health design practices. She has a background in behavioral science and qualitative methods. She conducted all qualitative interviews and led the data analysis. She had no prior relationship with the participants. The other researchers included 1 PhD student and 3 professors. The research team had expertise in implementation science TMFs (ARB; she/her), patient and caregiver engagement (KK; she/her), digital health (QP; she/her), and artificial intelligence (JP; he/him). All members of the research team were familiar with TMFs in digital health intervention design, but they had varying experiences when applying TMFs in practice.

### Participants

We recruited digital health design leaders who designed digital health interventions that aimed to support healthy behaviors in patients or the public. Design leaders had to be willing and able to comment on the use of TMFs in the digital health behavior change design process. Purposeful and snowball sampling was used to recruit participants. We used the results of our previous scoping review [15] and the expert knowledge of our research team to guide our purposive sampling. We aimed to recruit participants who designed digital health interventions for diverse health issues, resided in different locations, and had varying occupations across academic and industry settings. A detailed description of the participants is provided in the *Results* section and Multimedia Appendix 1. Design leaders who met the inclusion criteria were contacted through email. Our final interviewee sample size was determined using the notion of *conceptual depth*, where we stopped data collection when we perceived that we had produced sufficient richness in information related to our research questions [25]. Specifically, our research team had a reflexive discussion regarding the range of data we had collected, including its complexity, nuance, and whether we had enough data to tell a wider analytic story that would resonate with the literature and the concepts we had developed [25]. Reasons for nonparticipation included nonresponse and denial owing to previous publications on related topics.

### Data Collection

Semistructured interviews were conducted with design leaders using an interview guide with open-ended questions, allowing the interviewer to dig deeper into emerging topics [16]. The interview guide was based on the following question groupings: (1) What value do you see when using behavioral science TMFs in digital health design? (2) How do you incorporate TMFs in your own approach to digital health design? and (3) What do you think is needed to improve the design of digital health products? The content of the interview guide was finalized over several meetings with the research team and after running a pilot test with design leaders on a digital health team affiliated with an academic health science center in Ontario, Canada. Interviews were personalized based on the TMFs design leaders used in practice (ie, discussion about the Capability, Opportunity, and Motivation–Behavior model) and included reflections on other TMFs presented by the interviewer or interviewee. Single interviews were conducted using the Zoom software (Zoom Video Communications Inc). The interview questions lasted for approximately 30 to 45 minutes. Interviews were audio-recorded and transcribed using Otter.ai software. Notes were taken during the interviews by the lead researcher and recorded alongside the original transcripts.

### Data Analysis

We used an inductive thematic analysis approach to analyze interviewees’ experiences and perceptions. Specifically, we followed the codebook thematic analysis approach described by Braun and Clarke [23], which focused on coding reliability and reflexive thematic analysis [24]. In codebook thematic analysis, researchers develop and apply a coding frame to the data and conceptualize themes as topic summaries in relation to their research questions [24]. To conduct our codebook thematic analysis, we used a template codebook process, in which we developed a coding template based on a subset of our data and then applied this template to further data, while continuing to revise and refine the template [26]. This approach is not bound to any epistemological perspective and provides researchers with the flexibility to meet the needs of their studies [26]. The lead researcher (PV) began the analysis process by familiarizing herself with the data and rereading the interview transcripts and notes in detail. To generate the initial codes from the data, 3 members of the research team (PV, ARB, and KK) coded 2 transcripts each. The researchers discussed their findings with each other, paying close attention to how design leaders selected and used TMFs in their digital health design processes. PV organized these codes into meaningful clusters based on specific research questions. The larger research team then met to discuss the initial coding template and agreed on its descriptive and practical appropriateness. PV then coded the remaining transcripts using this initial template in NVivo 11 (QSR International), refining and modifying the template as necessary. After all the transcripts were coded, thematic topic summaries were generated in relation to the research questions. After these thematic topic summaries were agreed upon by a larger research team, an initial report of the qualitative analysis was produced. Participants were recontacted to review the written abstract and confirm their stated preference for recognition. As none of the participants requested any changes, no further modifications were applied to the report. The research team ensured trustworthiness through reflexive researcher notetaking, peer-to-peer debriefing, multiple-researcher triangulation, diagraming to make sense of coding connections, recontacting interviewees after the report was produced, and reporting direct quotes along with thematic topic summaries. This paper is reported in a way that aligns with the Consolidated Criteria for Reporting Qualitative Research checklist [27] and provides guidance for publishing qualitative research in informatics [28]. The presentation of the results is structured in a way that reflects our *codebook thematic analysis* approach, in which we have broken down the results into topic summaries that correspond directly to our research questions.

### Ethics Approval

All recruitment and data collection activities were approved by the University of Toronto Research Ethics Board (42515). Participants provided consent by either returning the signed consent form before the interview or by providing verbal consent at the beginning of the interview on a recorded line. To ensure that some design leaders were knowledge experts on the topic and wanted their intellectual contributions to be recognized, we obtained ethics approval that allowed interviewees to decide whether they wanted their name to be recognized or remain anonymous. Specifically, interviewees could have (1) their names attached to quotes, (2) their names recognized for their general contributions, or (3) their names kept anonymous. This decision was made after several discussions with our research team and potential interviewees regarding best practices when interviewing knowledge experts. We hope that this flexibility enhanced the validity of our study, because design leaders would feel more comfortable sharing their expert knowledge, knowing that they would be recognized in a way that aligned with their preferences.

## Results

### Participants

In total, 19 digital health design leaders were included in this study. Most interviewees requested to be recognized for their contributions to the study. Overall, our interviewees spanned multiple international locations, including 9 from North America, 8 from Europe, and 2 from Oceania. The interviewees were involved in designing digital health interventions that aimed to address a wide range of health concerns such as cardiovascular disease, diabetes, cancer, depression, joint pain, rheumatoid arthritis, multiple sclerosis, smoking addiction, and multiple chronic conditions. Interviewees were also involved in designing digital health interventions aimed at promoting a wide range of healthy behavior changes, such as increased physical activity, improved diet, medication adherence, smoking cessation, health self-advocacy, and mental wellness practices. Interviewees worked with diverse end user populations, such as older adults with chronic conditions, Black women with obesity, children and adolescents with depression, and indigenous communities with a distinctive understanding of health and well-being. Our interviewee sample included 10 interviewees who worked primarily in academia and 9 interviewees who worked primarily in industry. The interviewees consisted of 5 health researchers, 5 behavioral scientists, 3 clinicians, 2 scientific directors, 2 user-centered designers, 1 software engineer, and 1 business entrepreneur. However, it should be noted that most interviewees had proficiencies beyond their primary job title, and all were able to comment on TMFs in digital health behavior change design. A full description of the interviewees can be found in Multimedia Appendix 1. Preinterview demographic data (eg, gender and race-based data) were not collected from the interviewees. The limitations associated with the lack of preinterview demographic data are outlined in the *Discussion* section. The interpretations presented in this paper are the result of a qualitative analysis performed by our team of authors and are not a reflection of any single interviewee.

### The Value of TMFs in Digital Health Behavior Change Design

Design leaders had mixed feelings about the value of TMFs in digital health behavior change design. Leaders have suggested that we may need to reshape our views to see TMFs as a starting point rather than a final destination for evidence-informed design. Design leaders felt that TMFs can be a great foundation to obtain behavioral science on the agenda and to better conceptualize behavior change problems; however, there is much more to behaviorally informed design than simply relying on TMFs. TMFs may be most useful if we move beyond their limits and use them as a launching pad for a more interdisciplinary, user-centered design. Table 1 summarizes 6 important ways in which design leaders perceived that TMFs could add value to digital health behavior change design.

**Table 1:** How TMFs^a^ can add value to digital health behavior change design.

TMF value addition	Description	Illustrative quotations
1. TMFs add value by acting as a “gateway drug” to embed behavioral science	TMFs may be most valuable when used as mechanism to simplify complex behavioral science insights, demonstrating the applicability of behavioral insights in design.	“I became familiar with [the COM-B^b^ framework] and I started using it very heavily, because I feel like it’s a great gateway drug to behavioral science. It’s really easy for people to grasp if they don’t have behavioral science training, it layers well into how I’m working with UX^c^ professionals. You can organize design using COM-B and essentially execute really familiar activities if you’re a UX research professional. So it was easy for me to collaborate with my teammates. Using that framework, I kind of bring them along on the behavior science ride.” [Design leader, North America]
2. TMFs add value when they support health behavior conceptualization	TMFs may be most valuable when used to examine why people behave in certain ways in certain contexts, allowing us to better understand the problem before starting solutioning.	“(Using our model), we typically gain enough information to narrow down what we believe the barriers and facilitators are for behavior change. And that’s where the model leaves us. The model is a way for us to at the start of our behavioral science journey to very to make sure that no stone is left unturned. By the time we get to the ideation and the solution phase, we know that the barriers that we've identified are the right barriers.” [Design leader, North America]
3. TMFs add value when balanced with expert knowledge	TMFs may be most valuable when used alongside an expert in behavioral science who can appropriately apply concepts from the TMFs in practice.	“With the explosion a lot of popular books on psychology and behavioral science, it makes it really easy to think that it’s easy for anyone to change behaviors. But in practice it is actually super hard and super complex, and you have so many different factors to consider...I feel like I’m the expert gatekeeper that needs to protect my teams, and I only provide them what information [from TMFs] that they actually really need.” [Design leader, Europe]
4. TMFs add value when balanced with user-centered design principles	TMFs may be most valuable when balanced with user-centered design research principles, which push designers to dig deeper into users’ needs in their local settings.	“I think the most important thing (in digital health design) is a capacity to listen and a capacity to be curious about why people might be responding in a certain way...This real curiosity about each individual needs to be there. It is about understanding the user, their context, their goals, their strengths, and their weaknesses...I think there needs to be better alignment between the goals of the research and the goals of the people.” [Design leader, Holly Witteman, North America]
5. TMFs add value when they complement current design methods	TMFs may be most valuable when they complement current approaches and processes.	“I’m not really keen on introducing too much complexity most of the time. I rarely try to work in a different process. I try to understand the process they work with, and think about, okay, how in your process can we apply behavioral science?” [Design leader, Europe]
6. TMFs add value when they support both individual- and systems-level thinking	TMFs may be most valuable when they support thinking beyond the individual, exploring insights from implementation science and systems design.	“It is often a very comfortable place for behavioral science...to not necessarily deal with the full complexity of creating things with a systems-wide lens. A lot of the work I do is around choice infrastructure, which is sort of a new center of gravity. We focus on how do we take behavioral insights, use our models and frameworks, and find systematic ways to embed behavioral thinking into more system level conditions.” [Design leader, Ruth Schmidt, North America]

^a^TMFs: theories, models, and frameworks.

^b^COM-B: Capability, Opportunity, and Motivation–Behavior.

^c^UX: user experience.

### Considerations for Selecting and Applying the Most Value-Adding TMFs

Design leaders felt that there was considerable nuance in how a design team should select and apply the most value-adding TMF for their specific design needs. Leaders have suggested that the value of a TMF depends greatly on the setting and circumstances in which the TMF is applied:

Honestly, there is no one model that rules them all, there’s no one that’s perfect. Usually, one model has some form of limitations. But if [the design team] already has some form of knowledge, we usually have a discussion about “should we try using this or should we see this as a learning moment, and go to a model that is more advanced?” This [selection of TMF] is very context dependent, and there is no one way of approaching this because it might have to be done in different ways for different teams.

Design leader, Europe

Interviewees suggested several key questions to be considered when trying to select and apply a TMF to improve digital health behavior change design. We have summarized these questions into 12 groupings, which are listed in Textbox 1. These questions are fully detailed with illustrative quotations in Multimedia Appendix 2 and are further described in the text in the subsequent section.

**The 12 key questions for designers to consider about theories, models, and frameworks (TMFs) for digital health design.**

- TMF purpose: What purposes do you need the TMF for within your design process?
- TMF influence: How much influence does the TMF need to have on design decisions?
- TMF source: What setting did the TMF originate from and who developed the TMF?
- TMF appropriateness: Is the TMF the best way to facilitate meaningful solutioning?
- TMF complexity: How does the TMF balance comprehensiveness and simplicity?
- TMF accessibility: How easy is it for the design team to select and apply the TMF?
- TMF adaptability: How adaptable is the TMF to different processes and perspectives?
- TMF evidence: What effectiveness and relative advantage does the TMF have?
- TMF audience: Who will be using the TMF and what do they specifically need from it?
- TMF fit with team expertise: How will the TMF fit with experts present on the team?
- TMF fit with team mission: Is there enough capacity and motivation to use the TMF?
- TMF fit with external influences: Will using the TMF align with external pressures?

When questioning the *purpose of a TMF*, interviewees suggested it is important for designers to be clear and precise about what they need the TMF for (eg, is TMF required to guide the design progress, help conceptualize a behavior change issue, identify new design features and content, or evaluate how well an intervention is working in practice?). It is clear that the main purpose of using a TMF is to improve transparency in reporting. When questioning *the influence that a TMF will have*, interviewees suggested that designers should be aware of the differences between using a TMF to direct design decisions versus using a TMF to confirm design decisions. Although both approaches can be successfully used, it is helpful for designers to be reflexive of how much authority they give the TMF in guiding digital health design. When questioning *the source of a TMF,* interviewees suggested that it is important for designers to be thoughtful about whether the TMF was developed in a setting that is representative of the setting in which they are now using the TMF in. For example, some TMFs may present behavioral determinants based on the behavioral data of a specific population (eg, in populations with specific race, gender, socioeconomic class, or location distributions). Awareness of the limitations of TMF transferability is crucial. When questioning *the appropriateness of a TMF*, interviewees wanted designers to consider whether using it would expand or limit their creativity. For example, relying too much on the structure provided by a TMF may limit designers’ ability to truly listen to users regarding what they need and want in a solution. Understanding when it is appropriate to rely on insights from a TMF versus those from a user is an issue that designers should be reflexive about. When questioning *the complexity of a TMF*, interviewees had mixed feelings about whether it is better to use an intricate TMF that represents several viewpoints or a simple TMF that is easy to use in practice. Ultimately, interviewees suggested that the most suitable TMF complexity might be dependent on the design context (eg, the type of solution that needs to be designed) and the expertise of the design team (eg, whether there are behavioral scientists present). When questioning *the accessibility of a TMF*, it is important for designers to consider how easy it is to find, understand, and apply it in practice. An evidence-based TMF that is complicated to explain and difficult to decipher may not be the best for applied use in digital health behavior change design.

When questioning *the adaptability of the TMF*, interviewees suggested that digital health design is both a science and an art, and that the TMF needs to be flexible enough to allow design teams to layer on other approaches and tools. A TMF that does not complement the design team’s preferred practices and understanding may not be appropriate for selection. When questioning *the evidence of a TMF*, interviewees suggested that designers should be critical of whether a TMF has been shown to be effective in improving the design of comparable interventions in their context. In addition, interviewees wanted designers to consider whether the time, resources, and effort spent selecting, learning, and applying a TMF would provide a relative advantage compared with other approaches (eg, hiring a behavioral scientist or using another evidence-based approach). When questioning *the audience of the TMF*, the interviewees suggested that designers should consider who on their design team will use the TMF and what they will need out of it. For example, will the person using the TMF already be familiar with behavioral science or be completely new to the TMF concepts? When questioning *the fit of the TMF with team expertise*, it is important for design teams to consider what expertise they have in their team and what gaps the TMF fills. For example, if a team has little clinical expertise, then a TMF that represents more clinical considerations would be more appropriate. When questioning *the fit of the TMF with team culture and mission*, interviewees suggested that design leaders should consider whether introducing a TMF would be positive for team morale and trust. Design leaders should reflect on whether there is an appetite for behavioral science integration and the capacity for changes in team design processes. Finally, when *questioning the fit of the TMF with external influences*, interviewees suggested thinking practically about the design team’s bottom line and whether using an evidence-based TMF would improve profitability or applicability. Further information on these questions has been outlined in Multimedia Appendix 2.

### The Future of TMFs in Digital Health Design: Recommendations for Practice

Design leaders had several opinions about the future of TMFs in digital health behavior change design. Although design leaders agreed that having more comprehensive, easy-to-use TMFs would be valuable, they also felt that we may need to focus more on supporting design teams to build the capacity to integrate TMFs into their design practices. The design leaders’ recommendations for advancing TMF use in digital health design are summarized in Table 2.

**Table 2:** Recommendations to improve the value of theories, models, and frameworks (TMFs) moving forward.

Recommendation and description				Illustrative quotes
**1. Improve TMF reporting, design, and accessibility**				
	**1a. Improve the reporting of TMF use**			
		Design teams need to clarify exactly how they are applying certain TMFs in their design processes.	“I would be really curious how many people outside of academia actively use frameworks [to design digital health interventions]. I made a framework, but I don’t necessarily use it. I think it helps me organize my mental model of different intervention types, sure, and that’s useful. But I do wonder if anyone's ever used the behavior framework in practice.” [Design leader, North America]	
	**1b. Improve the design of TMFs**			
		TMFs should be comprehensive of multiple viewpoints, especially insights from implementation science and systems design	“For behavioral design in particular, I’m realizing more and more in my career that implementation has to be part of our view. We can’t just design the intervention anymore. So one of the things my team is working on right now is we're actually going to write up the implementation science aspect of our [digital health behavior change interventions].” [Design leader*,* North America]	
	**1c. Improve the accessibility of TMFs**			
		TMFs should be more accessible for design teams to select and input into their design process. Guidance on what tools to choose in certain situations may be helpful.	“I think the real opportunity lies in thinking through the [design thinking] phases, and thinking through what are the [behavioral science] activities within each one of those phases...What are the questions we need answered at every step of the way? And what are the tools, frameworks, techniques, templates that that we can use? You can rally around this as a design team. [Design leader, North America]	
**2. Improve design team capacity to use TMFs**				
	**2a. Improve design team structure**			
		Design teams may benefit from multidisciplinary membership so that TMFs concepts can be considered from multiple viewpoints.	“There’s a lot of design process phases where behavioral scientists will really need to bring in other people’s expertise. You see a lot of really terrible digital health products that are designed mostly by neuroscientists, academics, or behavioral scientists that don’t involve good service designers, UX^a^ designers, or UI^b^ designers.” [Design leader, Europe]	
	**2b. Improve design team training**			
		Design teams may benefit from training and consultancy ensure behavioral science thinking is embedded.	“[A consolidated framework] sounds useful from a simplified understanding of it. But maybe more fundamentally what needs to happen is that we need to have more expert behavioral scientists in the room working on behavior change problems and health challenges. And then fundamentally, further upstream, we need to be training more behavioral scientists and applied behavioral sciences.” [Design leader, North America]	
	**2c. Improve design team reflexivity**			
		Design teams may benefit from building in time for critical discussion, reflection, and evaluation of their own design methods.	“We don’t really like to do it where it’s like ‘the behavioral scientists do the work and then you hand it off to the human centered designer.’ We like to work in tandem, which can add a lot of frictions, because the way that we think is very different. And so we often are in conversations where I feel like we are going in circles, but actually we're having fundamental disagreements about how we think something should be done because our practices are very different. And so it’s sort of about having patience with this fact, because when you’re integrating multiple disciplines, it’s going to take more time. Being comfortable with it taking more time is important. And then when you actually test ideas out with the user base, you’re like, ‘wow, they actually they knew what they were doing.’” [Design leader, North America]	

^a^UX: user experience.

^b^UI: user interface.

## Discussion

### Principal Findings

This study presents a conceptualization of how TMFs are used to inform digital health behavior change design. Using a qualitative description approach, we conceptualized how design leaders perceive the value of TMFs, how they make decisions when selecting and applying TMFs, and what they think is needed to advance the utility of TMFs in the future.

**Figure 2:**
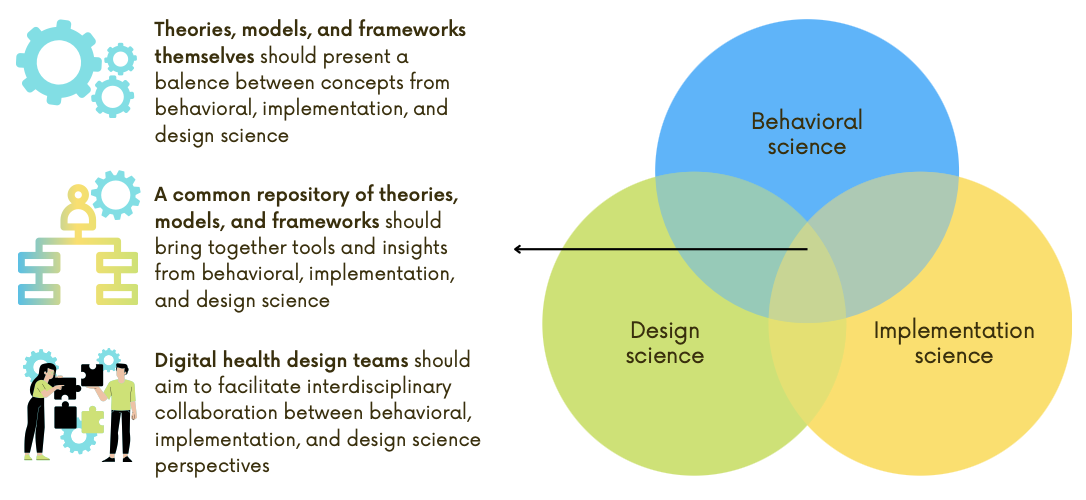
Advancing the value of theories, models, and frameworks in digital health behavior change design.

Regarding the value of TMFs in digital health behavior change design, this study found that design leaders had mixed feelings about the value TMFs currently provide. Although leaders shared several ways in which behavioral science TMFs helped inform their design practices, leaders questioned whether TMFs may be placing limitations on designing interventions that truly support health behavior change. Health behavior change does not simply arise from inputting evidence-based behavior change strategies into a digital health intervention [29,30]. An effective digital health behavior change intervention must also be designed with user-centered design principles as well as considerations for implementation and system design needs [31,32]. As the National Institute of Health’s Office of Behavioral and Social Sciences Research suggests, why invest in behavioral interventions if even the most evidence-based behavioral interventions are not adopted in practice [33]? Our results indicate that the future of digital health behavior change design requires a balance between insights from behavioral science, design science, and implementation science. Figure 2 presents how we can advance the value of TMFs in digital health behavior change design using the issues raised by design leaders.

Regarding the selection and application of TMFs, design leaders emphasized that there are many different factors that should be considered when choosing the most appropriate TMF for a specific design situation. One main issue that the field currently faces is that there are numerous behavioral science TMFs stored in different locations with little guidance on how to find and apply them [15]. This consolidation issue is not unique to this field; however, other disciplines have faced similar struggles with their TMFs [8]. For instance, the field of implementation science faced this problem and has made considerable progress in consolidating their wide range of TMFs [34]. The *Dissemination and Implementation Models in Health Web Tool* is an interactive web-based guide that helps practitioners understand the diverse range of TMFs in their field [34]. Work is also being conducted to develop a quality assessment tool that will help implementation science practitioners select and apply the most appropriate TMFs for their work [35]. The results of this study suggest that digital health behavior change design might benefit from similar advancements, which would bring together relevant TMFs into one repository with guidance on how practitioners can select and use these TMFs in digital health design practice. Future work might consider taking the list of questions presented in Textbox 1 and creating a decision tree that would help practitioners select TMFs that fit their design needs.

Finally, regarding the future of TMFs in digital health behavior change design, our results suggest that we need to not only to improve TMF design and accessibility but also improve the capacity of digital health design teams to meaningfully integrate TMFs in practice. Recent trends in digital health design suggest that there is increasing recognition of the value of leveraging behavioral insights in an interdisciplinary design team environment [36]. Sucala et al [36] recently published a paper that discussed the need for behavioral scientists to partner across different disciplines to ensure that their expertise is infused throughout digital health design. Rather than promoting a superficial use of behavioral insights, behavioral scientists need to better communicate the value of their deep expertise within interdisciplinary teams [36]. As of now, considerable effort is still being put into developing and disseminating taxonomies and toolkits for behavior change techniques. In academia, the Human Behavior Change Project works to consolidate peer-reviewed evidence on behavior change techniques [37]. In industry, MakeItToolkit provides insights into how design teams can translate behavior change techniques into intervention content and features [38]. However, none of these tools provide evidence-based guidance on how design teams should choose the right behavioral insights in specific digital health design settings and apply them in a way that appropriately meets users’ needs. Although behavioral science consultants may offer insight into how to do this on a project-to-project basis, there appears to be a gap in upstream training to embed behavioral science comprehension in digital health design more broadly. Not every design team member needs to be an expert in design science, behavioral science, and implementation science. However, it would be advantageous for design team members to comprehend and contribute to the critical application of these different insights in design practice. Future work might consider taking the issues discussed in this paper into consideration to develop upstream educational programming to ensure TMFs are used to their fullest capacity in digital health design projects.

### Implications for Future Research and Practice

This study offers a starting point for designers to think more deeply about the applicability of TMFs in digital health design practice. Researchers and practitioners may benefit from considering how the following recommendations might be applicable in their own context: (1) improving the reporting of TMF use in digital health design, paying close attention to how TMFs are being used to guide design process decisions; (2) improving the design of the TMFs to clearly yet comprehensively represent more viewpoints, especially from behavioral, implementation, and design science; (3) increasing the accessibility of TMFs, with a focus on how TMFs can be more easily selected and integrated into digital health design projects; (4) ensuring digital health design teams include experts from multiple different disciplines; (5) prioritizing upstream training of design team members to appreciate and understand the role of behavioral science alongside other knowledge bases; and (6) building in time for design teams to be reflexive and evaluate if their current design methods adequately facilitate the development of effectively engaging digital health interventions.

### Strengths and Limitations

The biggest limitation of this study relates to our omission of preinterview demographic data (eg, collecting gender- and race-based data). Although we aimed to recruit a diverse range of interviewees based on their location, occupation, and digital health design experience, we did not recruit them by using other demographic information. Even while attempting to recruit interviewees from different locations, it should be noted that our sample consisted only of participants from North America, Europe, and Oceania. The lack of participants from low-income, equity-deserving populations is notable. Digital health interventions are often critiqued for failing to account for the challenges faced by equity-deserving groups [39]. Although this study provides an important starting point for thinking more deeply about TMFs in digital health behavior change design, future research would benefit from taking an equity lens to this topic. Several interviewees pointed out that behavioral science TMFs have historically been built using behavioral data from predominantly White, high-income, and Western settings, which means that these TMFs may not be representative of the behavioral experiences in many equity-deserving populations. Many digital health behavior change interventions are designed to address health issues that disproportionality affects equity-deserving populations, yet the behavioral science TMFs we use to inform our digital health designs are often not reflective of the behavioral experiences of these groups. Future research that takes an equity lens on this topic may find that recommendations to improve the value of TMFs for digital health behavior change design are centered on equity-based issues. We hope that future research teams will take this into account when conducting more research on TMFs in digital health behavior change design.

It should also be highlighted that most of our design leader interviewees had a background in health services research or behavioral science and therefore carried certain assumptions about the applicability of evidence-based behavioral science TMFs in digital health design. Future research may benefit from purposely recruiting design leaders with expertise in different backgrounds. For example, our sample included only 3 clinicians, 2 user-centered designers, and 1 software engineer. Future work may find value in comparing and contrasting the perspectives and experiences of different stakeholders who use TMFs in digital health designs. Nonetheless, we believe that the information provided by our design leader sample has resulted in important, transferable insights that could help a diverse range of stakeholders who wish to use TMFs in their digital health design projects.

### Conclusions

With >90,000 digital health apps created in 2020 alone, there is concern that we may be entering an expensive period of trial and error, where digital health interventions are being rapidly designed with little intentionality [5,6]. Using evidence-based TMFs may be an important way to systematize the design of interventions that are more likely to support health behaviors. Nonetheless, selecting and applying the most value-adding TMFs appears to be challenging, and this might stagnate the integration of behavioral insights into digital health design. This study provides insight into how we may be able to extract more value from TMFs moving forward.

## Appendix

Multimedia Appendix 1 Participant descriptions.

Multimedia Appendix 2 Key questions for digital health designers about theories, models, and frameworks (TMFs).

## Abbreviations

TMFs: theory, model, and framework

## Data Availability

The data sets generated and analyzed during this study are available from the corresponding author upon reasonable request.
