# Supplementary material for: Making Sense of Theories, Models, and Frameworks in Digital Health Behavior Change Design: Qualitative Descriptive Study
Source: J Med Internet Res. 2023 Mar 15;25:e45095. doi: 10.2196/45095 (PMC10131681; doi:10.2196/45095)
Supplement: Multimedia Appendix 1 [file jmir_v25i1e45095_app1.docx]

**Multimedia Appendix 1: Participant descriptions.**

| **Design Leader Interviewees** | | | |
| --- | --- | --- | --- |
| **Name** | **Location** | **Type of Digital Health Project** | **Job Title** |
| Aline Holzwarth | USA | Multiple different types | Behavioral Science Lead, Apple, Health AI |
| Amelia Hyatt | Australia | Cancer Consultation Recording Application | Health Researcher, Peter MacCallum Cancer Centre |
| Amy Bucher | USA | Multiple different types | Chief Behavioural Officer, Lirio |
| Bart Pouls | Netherlands | Rheumatoid Arthritis Drug Adherence Application | Health Researcher and Pharmacist, Sint Maartenskliniek |
| Christian Jensen | Denmark | Musculoskeletal Pain Management Application | Software Engineer, Trade eXpansion |
| Dustin DiTommaso | USA | Multiple different types | Chief Design Office, meQuilibrium |
| Guido Giunti | Finland | Multiple different types | Physician and Digital Health Leader, University of Oulu |
| Holly Witteman | Canada | Multiple different types | Canada Research Chair in Human-Centred Digital Health |
| Luis Fernandez-Luque | Spain | Adaptive self-management for people living with chronic conditions | Chief Scientific Officer at Adhera Health |
| Lydia Sequeira | Canada | Mental Health and Suicide Prevention Applications | Health Researcher, Centre for Addictions and Mental Health and Canada Health Infoway |
| Malene Jagd Svendsen | Denmark | Musculoskeletal Pain Management Application | Health Researcher, University of Southern Denmark |
| Pauline Kabitsis | Canada | Multiple different types | Applied Behavioural Scientist, Common Thread |
| Robyn Whittaker | New Zealand | Multiple different types | Physician and Digital Health Researcher, National Institute for Health Innovation |
| Ruth Schmidt | USA | Multiple different types | Associate Professor at the Institute of Design (ID) |
| Samuel Salzer | Sweden | Multiple different types | Behavioral Science Advisor |
| Soren Kleberb | Denmark | Musculoskeletal Pain Management Application | CEO of SelfBack |
| Anonymous | USA | Physical Activity Application for General Wellbeing | Health Researcher at a University |
| Anonymous | Netherlands | Physical Activity Application for General Wellbeing | Health Researcher at a University |
| Anonymous | USA | Multiple different types | Director at Health Technology Research Centre |
