# Supplementary material for: Making Sense of Theories, Models, and Frameworks in Digital Health Behavior Change Design: Qualitative Descriptive Study
Source: J Med Internet Res. 2023 Mar 15;25:e45095. doi: 10.2196/45095 (PMC10131681; doi:10.2196/45095)
Supplement: Multimedia Appendix 2 [file jmir_v25i1e45095_app2.docx]

**Multimedia Appendix 2: Key questions for digital health designers about theories, models, and frameworks (TMFs).**

| **Question Grouping** | **Sub-Questions** | **Illustrative Quotes** |
| --- | --- | --- |
| 1. TMF Purpose: What purpose(s) do you need the TMF for within your design process? | 1.a. Do you need a TMF to *guide* the design process?  1.b. Do you need a TMF to help *conceptualize* behaviour change?  1.c. Do you need a TMFs to *identify* design features and content?  1.d. Do you need a TMFs to *evaluate* how your intervention is working? | 1.a. “I think drawing that line [using a model] is really useful because people often don't think about that until they start trying to design an intervention.” [Design leader, Europe]  1.b. “The framework is a way for us to at the start of our behavioral science journey to very to make sure that no stone is left unturned. Therefore, by the time we get to the ideation phase, and the solution phase, we really know that the barriers that we've identified are the right barriers. And so that model is the way to get us there. Then we sort of leave that model behind.” [Design leader, North America]  1.c. “I just think that [the BCT Taxonomy] helps us to talk to each other about what are the key ingredients of my program […] It just helps to compare different intervention options.” - Design leader, Oceania  1.d. “We also use the BCT Taxonomy to say, okay, so this is what a program that works looks like, this is what's behind it, these BCTs were used, and these constructs from theory drove how it was developed. So in order to really compare programs and figure out what works, the BCT Taxonomy can be really helpful.” [Design leader, Oceania] |
| 2. TMF Influence: How much influence does the TMF need to have on design decisions? | 2.a Do you need the TMF to direct/lead design decisions?  2.b. Do you need the TMF to confirm/check design decisions? | 2.a. “So we start our work [as behavioural scientists] very early in the design process. We identify our target behaviors, then we're mapping the determinants, then we use COM-B to categorize what those determinants are, then can bridge over into solutioning.” [Design leader, North America]  2.b. “I think our job [as behavioural scientists] is to be stewards of evidence, but we also need to be advocates for the patients and the end users. And we need to do our job nested within the requests, requirements, assumptions that clients have. Sometimes our clients are absolutely right and spot on, and we’re really just confirming ideas they already have.” [Design leader, Europe] |
| 3. TMF Source: What setting did the TMF originate from and who developed the TMF? | 3.a. Is the TMF representative of the population you are designing for?  3.b. Is it preferable to have a homegrown TMF or a pre-made TMF? | 3.a. “I very naively took the exact frameworks I used before and was like, well, we could just use them here. And then when we applied them, we very quickly realized that none of the solutions that we took from that framework resulted in anything fruitful” [Design leader, North America]  3.b. “Our framework came out of work that we did here […] We thought a lot about how to develop apps and how to implement them, and actually had a couple of failures which, you know, is really good thing to make you reflect on what you've done and haven't done well. So yeah, we developed our own framework after all these learnings.” [Design leader, Oceania] |
| 4. TMF Appropriateness: Is the TMF the best way to facilitate meaningful solutioning? | 4.a. Is the behavioural science TMF limiting or expanding solutioning?  4.b. Is the behavioural science TMF leading to user-centred solutioning? | 4.a. “It should be about moving beyond behavior change. [Behavioural science TMFs] are great, but also, by focusing too strongly on them, we may be leaving really important challenges out…”[Design leader, Ruth Schmidt, North America]  4.b. “There's a fundamental question in behavioral science I always find really fascinating. […] Should an intervention help you to develop skills that you don't currently have, or should it help you expand on or improve skills that you already have developed? What [behavioural designers] tend to do is address the first option. […] But, you know, there's more evidence for helping people build on their strengths […] That would be way better, but we tend to ignore that.” [Design leader, North America] |
| 5. TMF Complexity: How does the TMF balance comprehensiveness and simplicity? | 5.a Is the TMF comprehensive enough to represent multiple points of view?  5.b. Is the TMF simple enough to allow for ease of application in design? | 5.a. “The frameworks and models that we currently use, they're overly simplified. But they're simplified for a reason, so it's easier to apply them and easier for people to understand. But as we move into more complex areas of behavior change, and as we want to create greater change through our interventions, we really need to start using frameworks that include more, like more from the implementation sciences for example.” [Design leader, North America]  5.b. We work with a lot of research groups who often start off with an incredibly complex plan for what [their intervention] is going to look like. And these days, we're often finding that we just don't think it’s needed. It's huge amounts of complexity to add to the system, and it's more time, more money, more points of failure... It sounds like a really good idea from a behavior change theory point of view, but we're just not sure that it makes the difference that people think it's going to make. [Design leader, Oceania] |
| 6. TMF Accessibility: How easy is it for the design team to select and apply the TMF? | 6.a. How will the design team access the TMF?  6.b. How will external stakeholders access the TMF? | 6.a. “Our team has a lot of toolkits, but the thing is, unless you know about it [the TMF], and unless it’s presented in an easy way, it's really difficult to actually use it. Like there's so many [TMFs] out there and it will just become another resource.” [Design leader, North America]  6.b. “One thing I’ve observed [with our framework] is that it becomes very complicated to explain to a client what we’re doing. We need to figure out how to translate and present our framework back to non-specialists who are very, very busy people. It’s not like a model with 16 overlapping constructs is clean, linear, or understandable.” [Design leader, North America] |
| 7. TMF Adaptability:  How adaptable is the TMF to different processes and perspectives? | 7.a. How easy is it to balance the TMF with other design practices and approaches?  7.b. How easy is it to pair the TMF with other TMFs from different disciplines? | 7.a. “The application of theory is both science and art, and there is your leeway of deciding how you’re going to do this […] I think there needs to be a way that we can take participant feedback, and say, okay, what are they actually telling us and how does this align with something that we know works? How can we blend these two things, [evidence-based theory and user feedback], together.” [Design leader, Europe]  7.b. Creating new [TMFs] feels like a bit of an uphill battle because you have to dislodge what people are already familiar with. […] There's something in allowing people to bring their own tools and mindsets to the table, but finding a way to foster interoperability of how these perspectives come together. [Design leader, Ruth Schmidt, North America] |
| 8. TMF Evidence: What effectiveness and relative advantage does the TMF have? | 8.a. Is there proven evidence of effectiveness in the TMF improving design?  8.b. What relative advantage does using the TMF have over other approaches? | 8.a. “You could say [to a design team], your methods aren’t really having the lifts that you want and you're putting a lot of resources into these apps. So what if we do a trial […] and then we can say this is the effectiveness of your old design process and this is the effectiveness when you use the new method [with the TMF]. Then you can demonstrate its effect, and that would be a useful argument for design teams, right?” [Design leader, North America]  8.b. “[A consolidated framework] sounds useful from a simplified understanding of it. But maybe more fundamentally what needs to happen is that we need to have more expert behavioral scientists in the room working on behavior change problems and health challenges. And then fundamentally, like further upstream, we need to be training more behavioral scientists and applied behavioral sciences.” [Design leader, North America] |
| 9. TMF Audience: Who will be using the TMF and what do they specifically need from it? | 9.a. Who is going to use the TMF?  9.b. What do different stakeholders need from a TMF? | 9.a. “I keep coming back to who is the audience [of the TMF]? Who’s going to be using these? Is it someone who's really familiar with behavioural science? For many design teams, a checklist is not going to be that helpful if they don't have someone who knows what they're looking for in the first place.” [Design leader, North America]  9.b. “The Three-Delays Model was useful because it was a very simplistic model to show back to our clients and have them understand behavior change. But it actually wasn't very helpful for us when we were actually developing solutions, because it didn't unveil the specific barriers and enablers.” [Design leader, North America] |
| 10. TMF Fit with Team Expertise: How will the TMF fit with experts present on the team? | 10.a. How can a TMF help fill expertise gaps?  10.b. What role does a TMF play with expertise present? | 10.a. “Not every design team is going to have a technology expert, medical expert, design expert and behavioral expert. …The questions [that a checklist should ask] start with, okay, you don't have behavioral scientist, you should ask these questions. You don't have a tech person, well ask yourself these questions.” - Design leader, Europe  10.b. “Most of the time I'm actually not using a framework, I'm using my years of experience and expertise and asking, “what seems most relevant here based on talking to the customer”. […] When I look at the academic literature, I think no one is ever going to go so heavy into using [theories, models, and frameworks]. Like there are 96 BCTs? I know I'm not going to use that. […] These frameworks are both too much and not enough at the same time.” [Design leader, North America] |
| 11. TMF Fit with Team Culture and Mission: Is there enough capacity and motivation to use the TMF? | 11.a. Are there time and resources to learn and use a TMF?  11.b. Does the TMF align with team structure and goals? | 11.a. “There was room for testing things out and there was room for spending a year on back and forth between our different partners. […] When I think about the design process from our side, I think we learned a lot, and that's actually been a great experience for us.” [Design leader, Europe]  11.b. “Just the people who work there, the way we were organized, there was an appetite for behavioral science. And again, I think it was just built into the company in a way where nobody really felt threatened by that.” [Design leader, North America] |
| 12. TMF Fit with External Influences: Will using the TMF align with external pressures? | 12.a. How will using the TMF align with the external influences driving design decisions?  12.b. How will profitability/a business lens drive TMF use? | 12.a “Lots of design demands coming from a payer are really contrary to best practice. This is why I ask about money. Sometimes it doesn't matter if they want something bad [that doesn’t align with the evidence], if they want to pay for it, we’re going make something that goes against best practice. Because, you know, the goal at the end of the day is to make sure the company is alive, not to make the very best, evidence-based intervention.” [Design leader, North America]  12.b. “I actually consider three different types of frames that I'll need […] There’s a behavioral frame, which is like what is it that we're thinking about behaviorally. There's also a user frame, which is like what is realistic or relevant or viable for the humans involved? But then there's the business frame. If I come up with brilliant behavioral solution that works well for users, but it completely not viable from a financial or institutional perspective, it wont work.” [Design leader, North America] |
